# Optimising Soy and Pea Protein Gelation to Obtain Hydrogels Intended as Precursors of Food-Grade Dried Porous Materials

**DOI:** 10.3390/gels9010062

**Published:** 2023-01-12

**Authors:** Lorenzo De Berardinis, Stella Plazzotta, Lara Manzocco

**Affiliations:** Department of Agricultural, Food, Environmental and Animal Sciences, University of Udine, 33100 Udine, Italy

**Keywords:** plant proteins, heat gelation, gelling behaviour, structure, pH

## Abstract

Dried porous materials based on plant proteins are attracting large attention thanks to their potential use as sustainable food ingredients. Nevertheless, plant proteins present lower gelling properties than animal ones. Plant protein gelling could be improved by optimising gelation conditions by acting on protein concentration, pH, and ionic strength. This work aimed to systematically study the effect of these factors on the gelation behaviour of soy and pea protein isolates. Protein suspensions having different concentrations (10, 15, and 20% w/w), pH (3.0, 4.5, 7.0), and ionic strength (IS, 0.0, 0.6, 1.5 M) were heat-treated (95 °C for 15 min) and characterised for rheological properties and physical stability. Strong hydrogels having an elastic modulus (G′) higher than 10^3^ Pa and able to retain more than 90% water were only obtained from suspensions containing at least 15% soy protein, far from the isoelectric point and at an IS above 0.6 M. By contrast, pea protein gelation was achieved only at a high concentration (20%), and always resulted in weak gels, which showed increasing G′ with the increase in pH and IS. Results were rationalised into a map identifying the gelation conditions to modulate the rheological properties of soy and pea protein hydrogels, for their subsequent conversion into xerogels, cryogels, and aerogels.

## 1. Introduction

Xerogels, cryogels, and aerogels indicate dry porous materials produced by removing the solvent from a gel. Most studies have been carried out on the development of inorganic dried porous materials (e.g., silica and carbon-based) [1,2,3] to be used in a wide variety of applications, such as catalysis, environmental remediation, energy storage, and insulation [4,5,6,7]. Nevertheless, in recent years, growing interest has been focused on the development of biopolymeric-based dried porous templates, due to their biocompatibility, and non-toxic profile. Thanks to these characteristics, their application has been successfully extended to life science fields, including the biomedical and pharmaceutical sectors [8,9,10]. The potentialities of dried porous materials in the food sector are nowadays attracting large attention, due to their unique physico-chemical properties and techno-functionalities. Both cryogels and aerogels have been suggested as innovative delivery systems to protect bioactives and flavours during processing, storage, and digestion [11,12,13,14,15,16]. In addition, their capacity to absorb large amounts of food solvents has been identified as a key feature to modulate food structural properties [17,18]. For instance, they have been suggested as templates for oil structuring, leading to fat replacers with improved nutritional properties [16,19,20,21]. By contrast, as concerns xerogels, to the best of our knowledge, no applications in the food sector have been reported, despite the high potentialities of these materials have been demonstrated in other life science sectors.

To produce food-grade dried porous material, an aqueous gel is first produced by inducing the networking of the selected biopolymer in water, leading to a hydrogel [22]. To obtain a xerogel, subsequently, water is removed from the network by evaporative drying. The latter can also be performed by evaporating ethanol after substituting hydrogel water with ethanol [23,24]. The evaporative drying usually induces capillary forces during solvent removal, so that xerogels present low porosity [25]. Cryogels are instead obtained through freeze-drying, and thus by water sublimation [18]. This reduces the capillary forces, leading to materials with large pores and channels left upon the sublimation of water crystals grown during freezing [26]. Finally, aerogels are obtained by replacing the water contained in the starting gel with ethanol, followed by ethanol removal with a flow of CO_2_ in the supercritical state [27]. This technique preserves the structure of the initial network, and the dried material is thus characterised by low density and high internal surface area, due to the presence of micro- and macropores [28].

Food-grade xerogels, cryogels, and aerogels can be prepared either from polysaccharides or proteins. As concerns proteins, most literature studies focus on animal ones (e.g., whey, egg white, casein, gelatin) [29,30], while studies on the development of dried porous templates from plant proteins are limited to a few works exploiting silk fibroin, patatins, and soy proteins [31,32,33,34,35,36,37]. The interest in plant-based products is constantly growing due to their low environmental impact, low cost, and the possibility of being obtained from food industry wastes, in a circular economy perspective [38,39,40]. For these reasons, plant proteins are ideal candidates for developing sustainable dried porous materials intended as innovative ingredients for the food sector. However, the production of plant-based xerogels, cryogels, and aerogels is rather challenging. This is mainly due to the poor gelling properties of vegetable proteins compared to their animal counterparts. Protein gelation is commonly induced by heat treatment, during which the protein chains unfold, exposing their reactive groups, which subsequently drive protein reassembling in a 3D network. Although both covalent (i.e., S-S bridges) and weak interactions (i.e., hydrophobic interactions, hydrogen bonds, and electrostatic interactions) play an important role in the formation and stabilisation of protein gels [41], the availability of free -SH groups available for covalent stabilisation is known to lead to stronger gels. The possibility to obtain strong hydrogels is pivotal in determining their suitability in the conversion into dried porous materials, since the stronger the gel, the higher its capacity to structurally withstand the subsequent drying steps. In this regard, plant proteins present a lower number of -SH groups as compared to animal ones [42]. Moreover, the extraction process performed to isolate the protein fraction from the vegetable matrix, where it is intimately embedded in fiber–protein complexes, is known to induce structural modifications in the protein chains, further reducing gelling properties [43]. Nevertheless, several factors, including protein concentration, pH, and ionic strength, can be properly modulated to improve the plant protein gelling capacity. In this regard, the increase in protein concentration usually leads to a denser protein network, accounting for the formation of firmer gels that better maintain the original volume upon water removal [41]. When gelation occurs at a pH approaching the isoelectric point (pI), globular and strongly aggregated protein structures are formed, mostly driven by hydrophobic interactions [44,45]. At a pH far above or below the pI, instead, proteins form a fine-stranded network, as a result of the presence of surface charges which prevent intimate protein aggregation [46]. For example, aerogels derived from gels prepared near protein pI have been shown to present higher structural stability during drying, associated with lower density and higher pore sizes as compared to aerogels prepared far from the pI [28,47]. Gelation properties are also affected by ionic strength (IS). The increase in IS reduces electrostatic repulsive forces among protein chains, favouring the formation of a stronger network. For instance, the elastic modulus of pea protein gels was increased by 12 times by adding 0.3 M NaCl [41]. However, beyond a salt concentration threshold, specific for each protein (usually >2.0 M), a weakening of the hydrogel structure is commonly observed, due to salt-induced stabilisation of the protein structure, which suppresses protein unfolding during gelation [48,49].

This work aimed to systematically study the effect of gelation conditions on the physical properties of plant protein-based hydrogels, with the final aim of identifying the conditions leading to hydrogels suitable for the development of dried porous materials. For this purpose, soy and pea proteins were selected as the protein sources widely used as alternatives to animal proteins. Aqueous suspensions containing increasing amounts of soy and pea protein isolates (SPI and PPI) at different pH (3.0, 4.5 pI, 7.0) and IS (0.0, 0.6, 1.5 M) were heat-treated to induce gelation. The obtained hydrogels were characterised for rheological properties and physical stability, and the results were rationalised into a gelation map.

## 2. Results and Discussion

### 2.1. Effect of Protein Type and Concentration

SPI and PPI solutions were prepared at increasing concentrations from 10 to 20% (w/w) at pH 7.0, and thermally treated. Table 1 reports the appearance of the obtained SPI and PPI samples.

As expected, for both SPI and PPI, the increase in protein concentration resulted in a visible increase in system structuring [50,51]. At a given protein concentration, SPI always led to a more structured system as compared to PPI, so a minimum protein concentration of 15 and 20% (w/w) was required to form a semi-solid system by using SPI and PPI, respectively (Table 1). This difference was also confirmed by the rheological analysis. Supplementary Figure S1 reports the frequency sweep test results for SPI and PPI hydrogels obtained from 20% (w/w) protein solutions.

For both proteins, G′ higher than G″ and parallel to G″ was obtained, indicating the formation of gel systems [52]. The moduli of the PPI gel showed a higher frequency dependence (higher slope) than those of the SPI gels. The latter showed a negligible frequency dependence, indicating that a stronger gel structure was obtained; SPI gels also presented rheological moduli higher than those of the PPI gel, and a lower loss tangent (tan δ) (Table 1). These results confirm the higher gelling ability of SPI as compared to PPI. In agreement with the literature [53,54], this difference between SPI and PPI gelation properties can be attributed to the different compositions of the globulin fraction of the considered proteins. Soybean globulins are mainly represented by glycinin (11S) and β-conglycinin (7S), which present higher solubility than pea ones (legumin 11S and vicilin 7S). As a result, a higher protein fraction would remain homogeneously suspended during the gelation of soy proteins [43,53]. Moreover, soybean globulins have been previously demonstrated to present a threshold gelling concentration lower than pea ones [55].

The higher strength of the gel obtained with SPI rather than PPI was also related to an improvement in gel stability, as shown by the higher WHC values (Table 1). The increased density network obtained by increasing protein concentration was actually able to retain more water, due to the better distribution of the solvent in the 3D structure, as well as to the higher number of protein residues available for the interaction with water [56].

### 2.2. Effect of pH

The precursor protein solutions were adjusted to pH 3.0, 4.5, and 7.0 and thermally treated. Independently of the pH, self-standing gelled systems were only obtained at 15 and 20% (w/w) SPI concentrations and at a 20% (w/w) PPI concentration. As representative examples, Table 2 reports the appearance and the rheological parameters of the hydrogels obtained from the SPI and PPI solutions at 20% (w/w) protein concentration and adjusted at the different pH values.

Similar to data achieved at pH 7.0 (Table 1), also at pH 3.0 and 4.5, SPI led to higher system structuration as compared to PPI. At pH 4.5, which is close to protein pI, a particulate gel, otherwise known as a microgel, was obtained with both proteins [57,58]. Proteins actually show a higher tendency towards aggregation in the isoelectric region, where the net charge is low, and thus protein–protein interactions are promoted with the formation of spherical particles, which, at a high protein concentration, can randomly associate into larger self-supporting hydrogels [58]. By contrast, at pH values away from the pI, where strong electrostatic repulsions are present, the gels present a fine-stranded structure.

For both proteins, the decrease in pH from 7.0 (Table 1) to 3.0 (Table 2) caused a significant decrease in system structuration, as evidenced by the rheological parameters. In fact, not only both moduli showed lower values for gels prepared at pH 3.0 as compared to those obtained at neutral pH, but they also presented a slightly higher frequency dependence. In this regard, Supplementary Figure S2 shows the effect of the pH change on the frequency sweep results of PPI gels prepared at 20% (w/w) protein concentration at pH 3.0 and 7.0. A significant decrease in gel strength was instead observed upon adjusting the protein solution at pH 4.5 (Table 1 and Table 2). This can be attributed to the different microstructure of the hydrogels obtained at different pHs. In particular, microgelled systems obtained near the pI are stabilised by weak surface interactions among spherical protein aggregates, which can easily flow one on the other [59]. By contrast, at pHs far from the pI (pH 3.0 and 7.0), stranded gel structures are obtained, stabilised by numerous disulphide bridges and weak-interaction entanglement regions, thus accounting for the higher resistance to mechanical perturbation [58]. Moreover, in the isoelectric region, protein solubility is minimised, resulting in a significant decrease in well-solubilised protein fractions able to efficaciously interlink in a 3D gel network [53].

For both SPI and PPI, pH had a negligible effect on gel stability, as indicated by the comparable WHC values (Table 1 and Table 2). This is probably due to the counterbalancing effect of the high protein concentration on the effect of pH. In other words, the effect of the different gel architectures induced by pH would be made negligible in the presence of a high protein concentration, which would increase the network density, thus allowing a high solvent retention [53].

### 2.3. Effect of Ionic Strength

The precursor protein solutions were added with different NaCl amounts to modulate the ionic strength (IS) of the system. As representative examples of the effect of this parameter at low protein concentrations, Table 3 shows the appearance of systems obtained upon the thermal treatment of 10% (w/w) SPI and 15% (w/w) PPI solutions, at pH 7.0, and having 0.6 and 1.5 M IS.

Although the final system showed an evident phase separation, as compared to the system with no salt added (Table 1), which showed a liquid-like homogeneous structure, the increase in IS resulted in a local gelling effect with the formation of a microgel-like structure. This effect can be traced back to the shielding effect of salt ions of the protein surface charge, favouring protein aggregation [60]. The positive effect of the IS increase on SPI and PPI gelling properties was also observed at a higher protein concentration. In this regard, Table 3 reports the appearance and the rheological parameters of the hydrogels obtained from 20% (w/w) SPI and PPI solutions at pH 7.0, at 0.6 and 1.5 M IS. As compared to the gels obtained without salt addition (Table 1), the increase in IS resulted in particulate gels, well-evident in the case of the PPI-based systems (Table 3). This was due to the changes induced by the increase in IS in the gel microstructure, which shifted from a fine-stranded structure (low IS) to a particulate structure (high IS) [22]. NaCl concentration increase also caused a considerable increase in both SPI and PPI gel strength, as indicated by the increase in G′ values (Table 1 and Table 3), as shown in Supplementary Figure S3, which reports the frequency sweep results for PPI gels at 20% (w/w) protein concentration at 0.0 and 1.5 M IS. The presence of Na^+^ ions actually promotes protein–protein interactions during gelation, due to the reduction of the repulsive electrostatic interactions between protein chains [51]. Moreover, the increase in IS is known to promote the so-defined “salting-in” effect, i.e., the increase in the solubility of globulins, which are the main protein fraction of both SPI and PPI [61]. A higher IS thus results in higher availability of well-hydrated proteins available for networking during gelation [51,62].

IS also affected gel stability. In the case of the SPI gels, WHC decreased with IS, despite the higher gel strength (Table 3). Similar results were found for gels from both soy [63,64] and egg white proteins [65,66,67,68] and can be attributed to the microstructural changes induced by the presence of ions. In this regard, Munialo et al. [69] have demonstrated that a gel with an evenly distributed fine-stranded network, obtained at low IS, generally presents higher WHC as compared to particulate gels, obtained at high IS, where water is less tightly trapped. Likewise, Maltais et al. [70] and Urbonaite, et al. [71,72] reported an inverse correlation between aggregate size and WHC, with larger aggregates resulting in lower WHC. On the contrary, in the case of PPI hydrogels, the increase in IS promoted an increase in the WHC. It can be inferred that, in this case, the increased gel structural properties obtained upon NaCl addition (Table 1 and Table 3) prevailed over the microstructural changes induced by the IS increase.

### 2.4. Gelation Map

**Figure 1:**
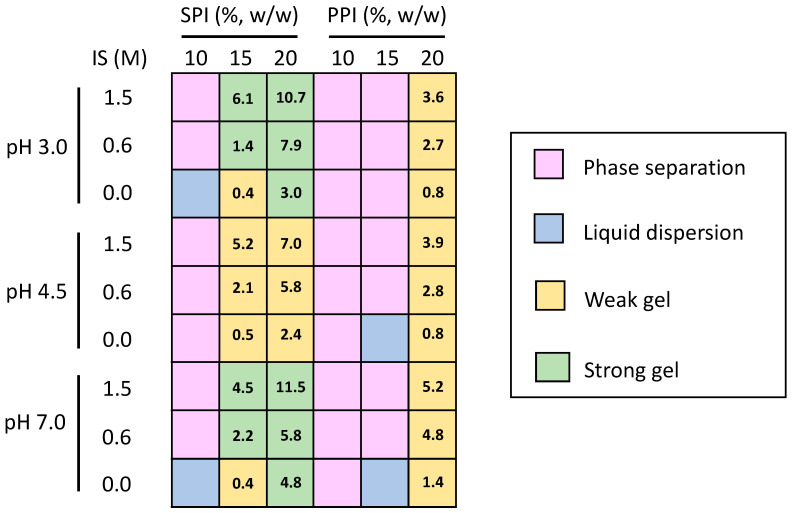
Gelation map of soy (SPI) and pea protein isolate (PPI) at increasing protein concentration (%, w/w), pH, and ionic strength (IS). The mean values of elastic modulus (G′ × 10^3^ Pa) of the gelled systems are also reported within cells.

Collected data were further elaborated and rationalised in order to obtain a gelation map (Figure 1), which is useful to have an immediate view of the gelation performances of SPI and PPI under the considered conditions.

The obtained map clearly highlights the complex effect of protein type, pH, IS, and their combination on the sample structure. For example, the higher gelation propensity of SPI as compared to PPI is immediately visible, as well as the higher structuration obtained far away from the protein isoelectric region pH or increasing the IS. This map represents a useful tool to identify optimal conditions leading to SPI and PPI gels presenting the desired physical properties. In particular, the conditions allowing for the preparation of hydrogels presenting a network strong enough to withstand the conversion into xerogels, cryogels, and aerogels can be identified. Moreover, additional considerations can be drawn, with the aim of optimising the production process of these dried porous materials. For example, at pH 3.0 or 7.0, in view of minimising the consumption of SPI, and thus raw material costs, while also maintaining a strong gel structure, the possibility to reduce the SPI concentration from 20 to 15% (w/w) while increasing the ionic strength can be identified. Similarly, in the case of PPI, it is immediately evident how only weak gels can be obtained at 20% concentration.

## 3. Conclusions

The results collected in this study show that the gelling behaviour of vegetable proteins is highly dependent on both the protein nature and formulation parameters (protein concentration, pH, ionic strength). In particular, hydrogel strength can be enhanced by choosing soy proteins over pea ones, as well as avoiding the isoelectric region and increasing the ionic strength. The obtained gelation map can be considered a useful tool to identify the optimal conditions to produce soy and pea protein hydrogels with physical properties suitable for the subsequent conversion into xerogels, cryogels, and aerogels.

The results obtained in this research, although relevant to soy and pea protein isolates solely, clearly indicate the potential of plant proteins as interesting precursors for the production of food-grade and plant protein-based dried porous materials. Further studies are therefore required to investigate the correlation between the physical and techno-functional properties of the precursor hydrogel and the resulting dried materials. In this regard, different drying processes such as evaporative drying, freeze-drying, and supercritical drying can be applied to convert the obtained hydrogels into xerogels, cryogels, and aerogels, respectively. At the same time, a comprehensive characterisation of the dried templates obtained thereof could be performed. The latter should include the physical characterisation of the materials (e.g., SEM microstructure, BET surface area, porosity) but also their interaction properties with food fluids (oil, water) to obtain a first insight into their applicability as innovative food ingredients.

## 4. Materials and Methods

### 4.1. Soy and Pea Protein Solution Preparation

Aqueous solutions presenting different ionic strength (IS), 0.6 and 1.5 M, were prepared by adding NaCl (Sigma Aldrich, Milan, Italy) in deionised water (System advantage A10^®^, Millipore S.A.S, Molsheim, France). Deionised water without the addition of NaCl was considered to have an IS equal to 0.0 M. Aqueous solutions were added with 10, 15, or 20% (w/w) of soy (SPI) or pea (PPI) protein isolates (Myprotein, Manchester, England). The suspensions were subjected to high shear mixing at 1120× *g* for 1 min (Polytron PT-MR3000, Kinematica AG, Littau, Switzerland), and pH was adjusted to 3.0, 4.5, and 7.0 by adding 1 M NaOH or HCl.

### 4.2. Heat Treatment

To induce gelation, soy and pea protein suspensions were transferred in 50 mL-sealed falcon tubes and subjected to thermal treatment in a water bath (95 °C for 15 min), followed by cooling in an ice bath (0 °C for 15 min). The heat-treated samples were then stored at 4 °C for 48 h, until analysis.

### 4.3. Image Acquisition

Images were captured with a digital camera (EOS 550D, Canon, Milano, Italy) in an image acquisition cabinet (Immagini & Computer, Bareggio, Italy). The digital camera was positioned in an adjustable stand positioned at 45 cm from the samples and enlightened by 4 × 100 W frosted photographic floodlights, in a position allowing minimum shadow and glare.

### 4.4. Rheological Properties

Hydrogel rheological properties were tested using an RS6000 Rheometer (Thermo Scientific RheoStress, Haake, Germany), equipped with a Peltier system for temperature control. The analysis was performed with a parallel plate geometry, with a gap of 2.0 mm at 20 °C. Hydrogels were cut into cylinders with 2 mm of height and 20 mm of diameter. The linear viscoelastic region (LVR) was determined using an oscillatory sweep test (0.01 to 1000 Pa at 1 Hz frequency). The frequency sweep tests were carried out increasing the frequency from 0.1 to 20 Hz, at stress values selected in the LVR.

### 4.5. Physical Stability

The physical stability of hydrogels was evaluated based on their water-holding capacity (*WHC*). Hydrogels were accurately weighed (*W*_1_) and transferred into 1.5 mL-Eppendorf microcentrifuge tubes, and then centrifugated at 15,000× *g* for 15 min at 4 °C (D3024, DLAB, Scientific Europe S.A.S, Schiltigheim, France). The supernatant was then removed, and the samples were weighed again (*W*_2_). The *WHC* was determined according to Equation (1).

(1) $$WHC=\frac{W_{1}-\left(W_{1}-W_{2}\right)}{W_{1}}\cdot 100$$

### 4.6. Data Analysis

Data are expressed as the mean ± standard deviation of at least three measurements resulting from two replicates. The statistical analysis was performed using the program R version 4.1.2 (The R Foundation for Statistical Computing, Vienna, Austria). The homogeneity of the variance was evaluated with Bartlett tests, a one-way ANOVA was applied, and the difference between the averages was assessed by the post-hoc Tukey test (*p* < 0.05).

## Figures and Tables

**Table 1:** Appearance, elastic (G′), loss modulus (G″), loss tangent (tan δ), and water-holding capacity (WHC) of soy protein isolate (SPI) and pea protein isolate (PPI) systems obtained after heat treatment of protein solutions at 10, 15, and 20% w/w; at pH 7.0; and 0.0 ionic strength.

Protein	Concentration (%, w/w)	Appearance	G′ × 10^2^ (Pa)	G″ × 10^2^ (Pa)	Tan δ	WHC
SPI	10	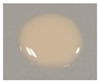	N.D.	N.D.	N.D.	N.D.
	15	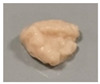	3.94 ± 0.24 ^c^	0.77 ± 0.04 ^c^	0.19 ± 0.01 ^b^	89.51 ± 3.56 ^a^
	20	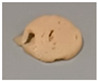	47.57 ± 1.61 ^a^	6.60 ± 0.24 ^a^	0.14 ± 0.01 ^c^	99.60 ± 0.44 ^a^
PPI	10	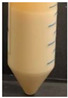	N.D.	N.D.	N.D.	N.D.
	15	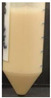	N.D.	N.D.	N.D.	N.D.
	20	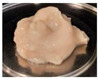	13.64 ± 0.24 ^b^	3.13 ± 0.05 ^b^	0.23 ± 0.01 ^a^	75.19 ± 0.03 ^b^

N.D.: not determined, since the system did not gel. ^a, b, c^: means indicated by different letters in the same column are significantly different (*p* < 0.05).

**Table 2:** Appearance, storage modulus (G′), loss modulus (G″), loss tangent (tan δ), and water-holding capacity (WHC) of soy protein isolate (SPI) and pea protein isolate (PPI) hydrogels at 20% protein concentration at pH 3.0 and 4.5, and 0.0 ionic strength.

Protein	pH	Appearance	G′ × 10^2^ (Pa)	G″ × 10^2^ (Pa)	Tan δ	WHC
SPI	3.0	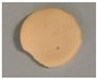	30.14 ± 2.78 ^a^	4.10 ± 0.46 ^a^	0.14 ± 0.01 ^c^	99.80 ± 0.09 ^a^
	4.5	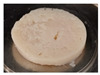	24.32 ± 0.66 ^b^	3.36 ± 0.85 ^b^	0.14 ± 0.01 ^c^	99.68 ± 0.07 ^a^
PPI	3.0	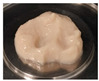	8.52 ± 1.26 ^c^	2.38 ± 0.31 ^c^	0.28 ± 0.01 ^a^	67.07 ± 0.73 ^b^
	4.5	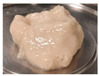	7.89 ± 0.59 ^d^	1.99 ± 0.73 ^d^	0.25 ± 0.01 ^b^	75.00 ± 2.99 ^b^

^a, b, c, d^: means indicated by different letters in the same column are significantly different (*p* < 0.05).

**Table 3:** Appearance, storage modulus (G′), loss modulus (G″), loss tangent (tan δ), and water-holding capacity (WHC) of soy protein isolate (SPI) and pea protein isolate (PPI) hydrogels at 10, 15, or 20% (w/w) protein concentrations at 0.6 and 1.5 M ionic strength.

Protein	Concentration (%, w/w)	Ionic Strength (M)	Appearance	G′ × 10^2^ (Pa)	G″ × 10^2^ (Pa)	Tan δ	WHC
SPI	10	0.6	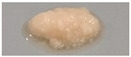	N.D.	N.D.	N.D.	N.D.
		1.5	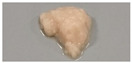	N.D.	N.D.	N.D.	N.D.
	20	0.6	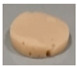	58.21 ± 7.10 ^b^	9.97 ± 1.19 ^b^	0.17 ± 0.01 ^b^	97.95 ± 0.70 ^a^
		1.5	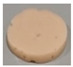	115.51 ± 46.08 ^a^	22.40 ± 8.52 ^a^	0.19 ± 0.01 ^a^	89.24 ± 3.17 ^b^
PPI	15	0.6	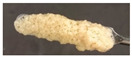	N.D.	N.D.	N.D.	N.D.
		1.5	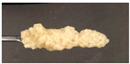	N.D.	N.D.	N.D.	N.D.
	20	0.6	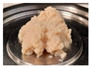	48.48 ± 0.29 ^b^	11.52 ± 0.07 ^b^	0.24 ± 0.01 ^c^	84.76 ± 3.56 ^b^
		1.5	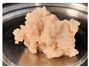	51.57 ± 6.38 ^b^	12.56 ± 1.64 ^b^	0.24 ± 0.01 ^c^	91.96 ± 0.02 ^c^

N.D.: not determined, since the system did not gel. ^a, b, c^: means indicated by different letters in the same column are significantly different (*p* < 0.05).

## Data Availability

Data available on request to the corresponding author.

## Supplementary Materials

The following supporting information can be downloaded at: https://www.mdpi.com/article/10.3390/gels9010062/s1, Figure S1: Elastic (G′) and viscous (G″) modulus of soy (SPI) and pea (PPI) hydrogels obtained from 20% (w/w) protein solutions. Figure S2. Elastic (G′) and viscous (G″) modulus of pea protein isolate (PPI) hydrogels obtained from 20% (w/w) protein solutions at pH 3.0 and 7.0. Figure S3. Elastic (G′) and viscous (G″) modulus of pea protein isolate (PPI) hydrogels obtained from 20% (w/w) protein solutions at 0.0 and 1.5 M ionic strength.
