# Supplementary material for: Optimising Soy and Pea Protein Gelation to Obtain Hydrogels Intended as Precursors of Food-Grade Dried Porous Materials
Source: Gels. 2023 Jan 12;9(1):62. doi: 10.3390/gels9010062 (PMC9858295; doi:10.3390/gels9010062)
Supplement: Supplementary file 1 [file gels-09-00062-s001.zip › gels-2132584-supplementary.pdf]

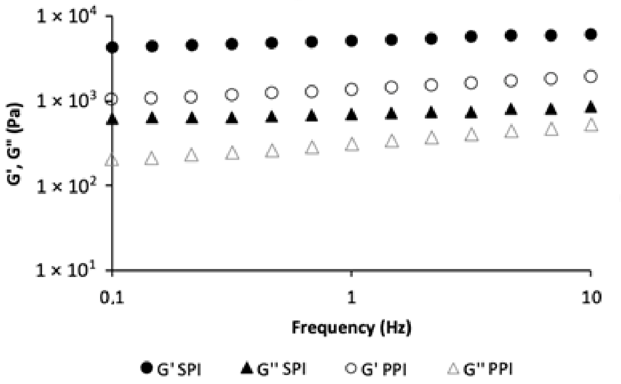

Figure S1. Elastic (G′) and viscous (G″) modulus of soy (SPI) and pea protein isolate (PPI) hydrogels obtained from 20% (w/w) protein solutions.

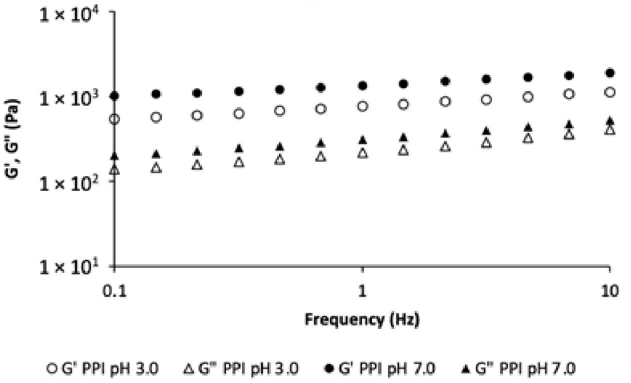

Figure S2. Elastic (G′) and viscous (G″) modulus of pea protein isolate (PPI) hydrogels obtained from 20% (w/w) protein solutions at pH 3.0 and 7.0.

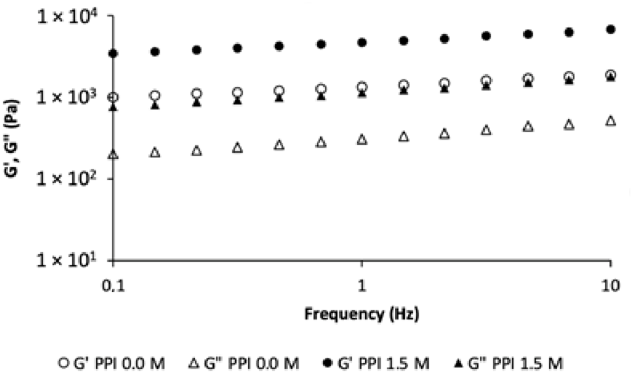

Figure S3. Elastic (G′) and viscous (G″) modulus of pea protein isolate (PPI) hydrogels obtained from 20% (w/w) protein solutions at 0.0 and 1.5 M ionic strength.
